# Supplementary material for: Unraveling the origin of air-stability in single-crystalline layered oxide positive electrode materials
Source: Nat Commun. 2025 Jul 15;16:6519. doi: 10.1038/s41467-025-61304-0 (PMC12263995; doi:10.1038/s41467-025-61304-0)
Supplement: Supplementary file 1 — Supplementary Information [file 41467_2025_61304_MOESM1_ESM.pdf]

Supplementary information

Unraveling the origin of air-stability in single-crystalline layered oxide positive electrode materials

Lei Yu[1#], Jing Wang[2#], Tao Zhou[1], Weiyuan Huang[2], Tianyi Li[3], Lu Ma[4], Xianghui Xiao[4], Seoung-Bum Son[2], Steven N. Ehrlich[4], Jianguo Wen[1], Khalil Amine[2*], Tongchao Liu[2*]*

[1]Center for Nanoscale Materials, Argonne National Laboratory, Lemont, IL, 60439, USA

[2]Chemical Sciences and Engineering Division, Argonne National Laboratory, Lemont, IL, 60439, USA

[3]X-ray Science Division, Advanced Photon Sources, Argonne National Laboratory, Lemont, IL, 60439, USA

[4]National Synchrotron Light source II, Brookhaven National Laboratory, Upton, NY 11973, USA

[#]These authors contributed equally to this work.

*Corresponding author: jwen@anl.gov (J. W.); amine@anl.gov (K. A.); liut@anl.gov (T. L.);

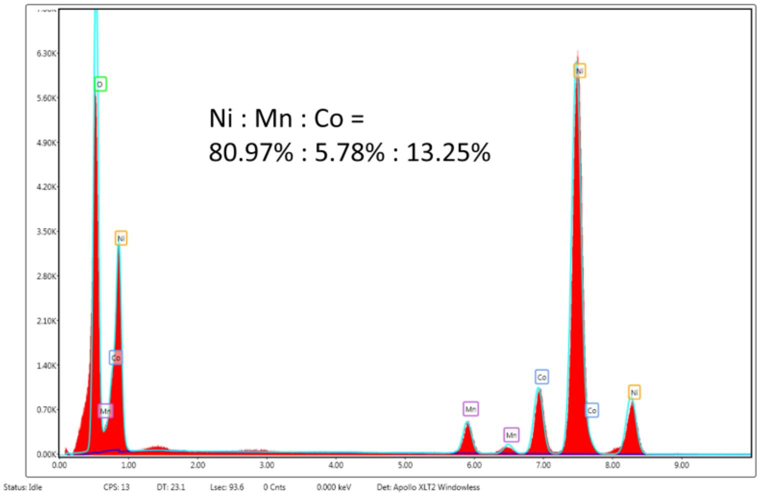

Supplementary Fig. 1 | EDS spectrum of pristine SC81 sample.

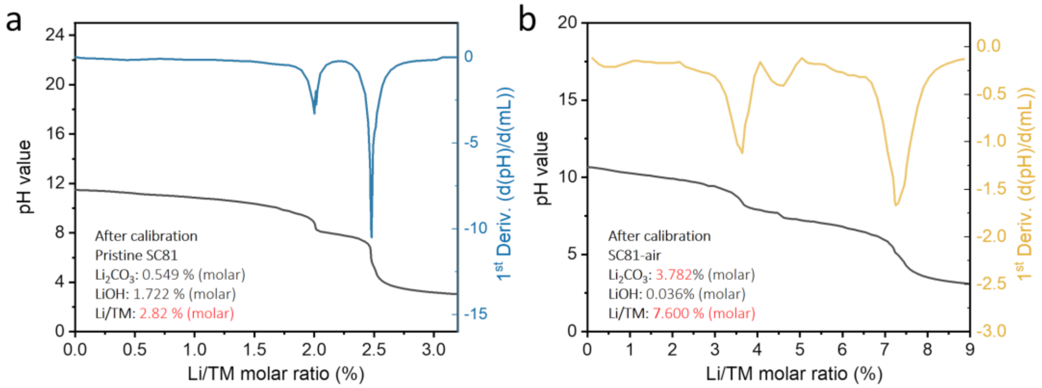

Supplementary Fig. 2 | Titration curves of (a) pristine SC81 and (b) SC81-air samples.

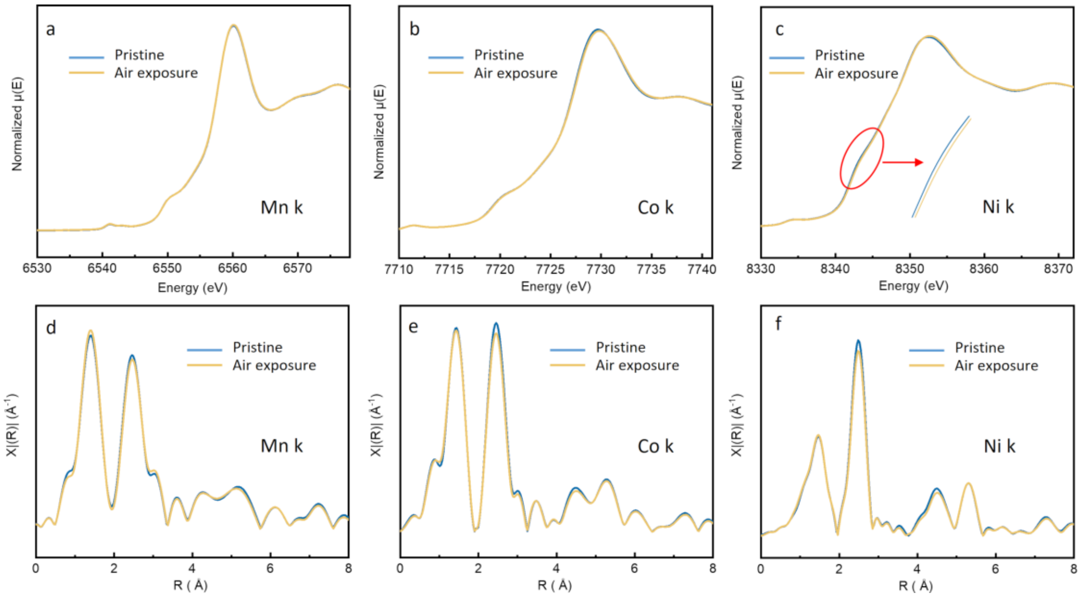

Supplymentary Fig. 3 | XAS comparison between pristine SC81 and SC81-air at K edges of Mn, Co and Ni: **a-c**, normalized X-ray absorption near-edge structure spectra, **d-f**, extended X-ray absorption fine structure spectra.

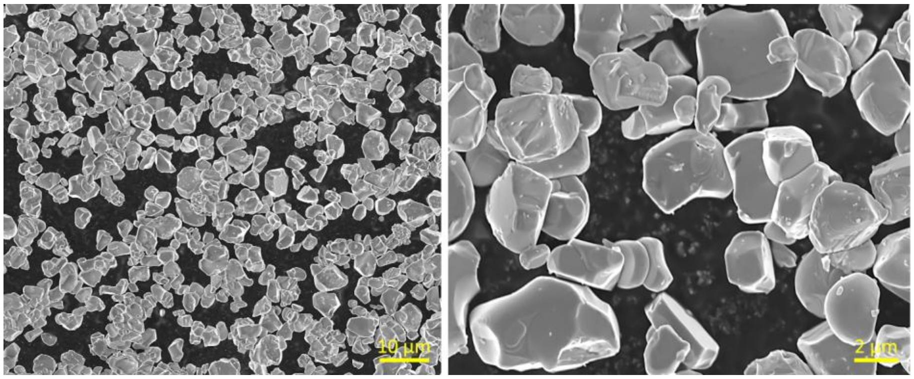

Supplymentary Fig. 4 | SEM iamges of pristine SC81.

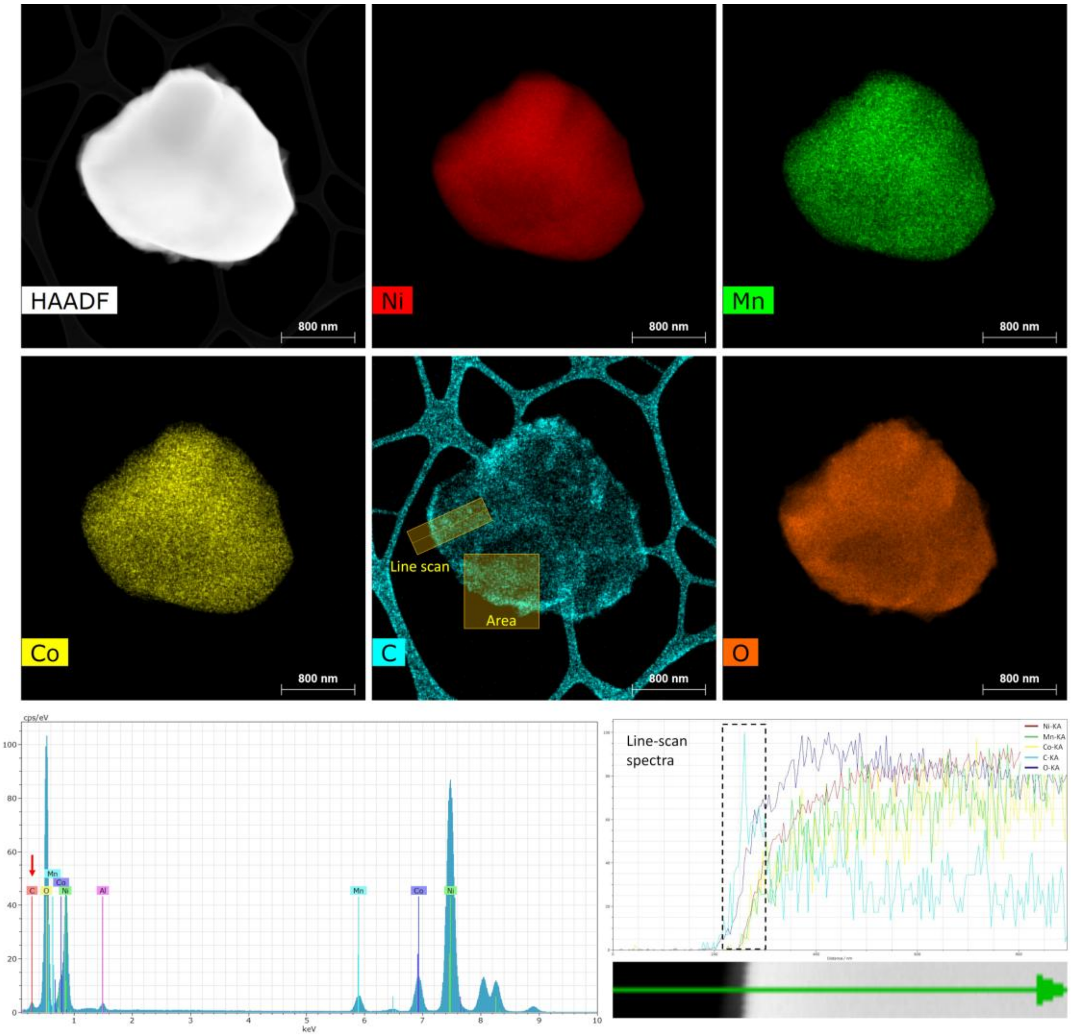

Supplymentary Fig. 5 | EDS characterization of the SC81-air sample.

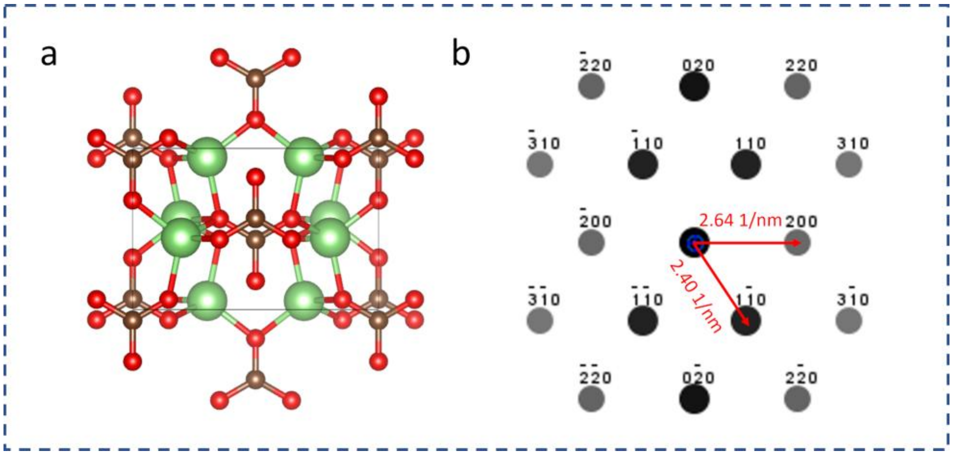

Supplymentary Fig. 6 | a, Structural model of the Li_2CO_3 phase (*C*2/*c* space group), where green spheres represent Li atoms; red spheres represent O atoms; brown spheres represent C atoms. **b**, Simulated diffraction pattern under the zone axis of [001].

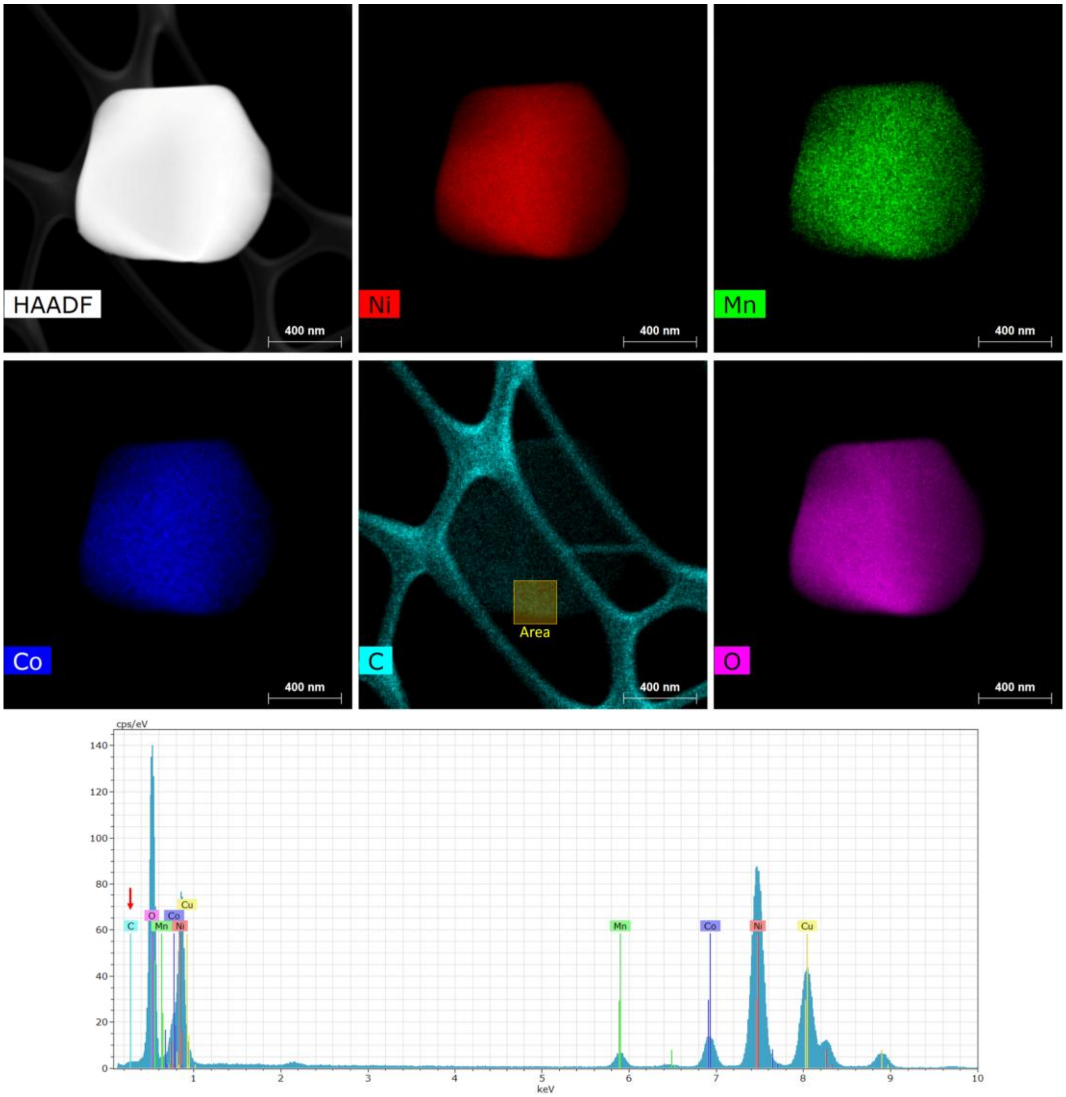

Supplymentary Fig. 7 | EDS characterization of the discharged SC81-air sample after 5 cycles at a specific current of 0.1C (20 mA g^{-1}) and a temperature of 25 ± 1 °C within the voltage ranges of 2.8–4.5 V.

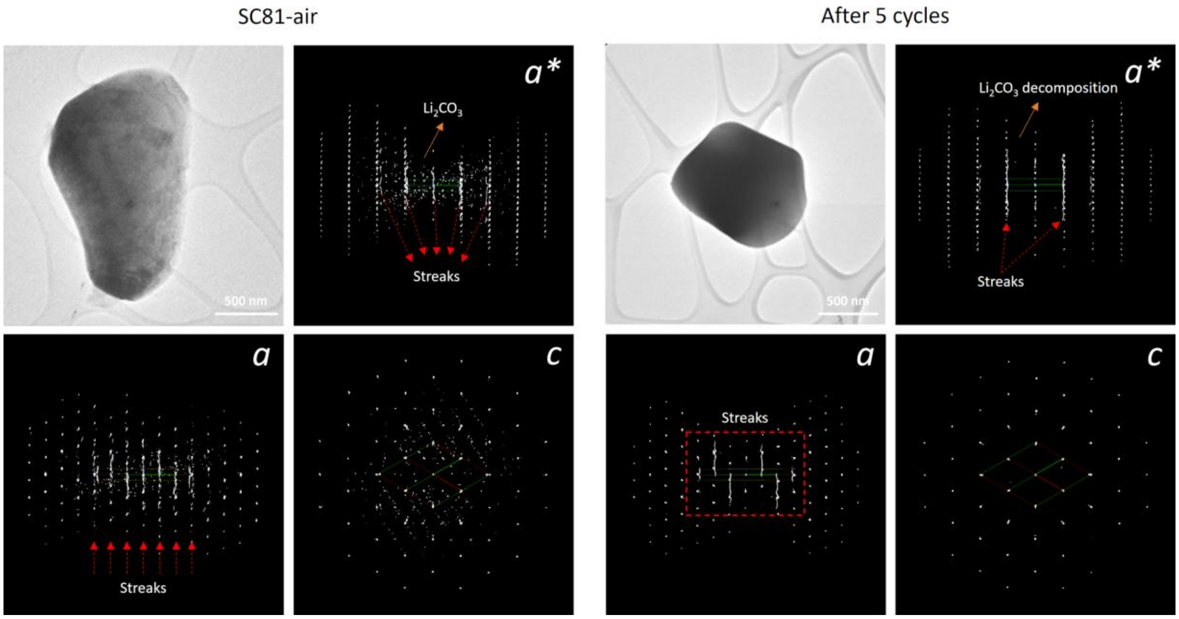

Supplymentary Fig. 8 | The 3D-CRED results of SC81-air particles before and after 5 cycles at a specific current of 0.1C (20 mA g^{-1}) and a temperature of 25 ± 1 °C within the voltage ranges of 2.8–4.5 V. The disordered diffraction points represent the polycrystalline nature of Li_2CO_3 surface impurity. The streak diffraction indicates the presence of lattice distortion and stacking defects in the layered structure.

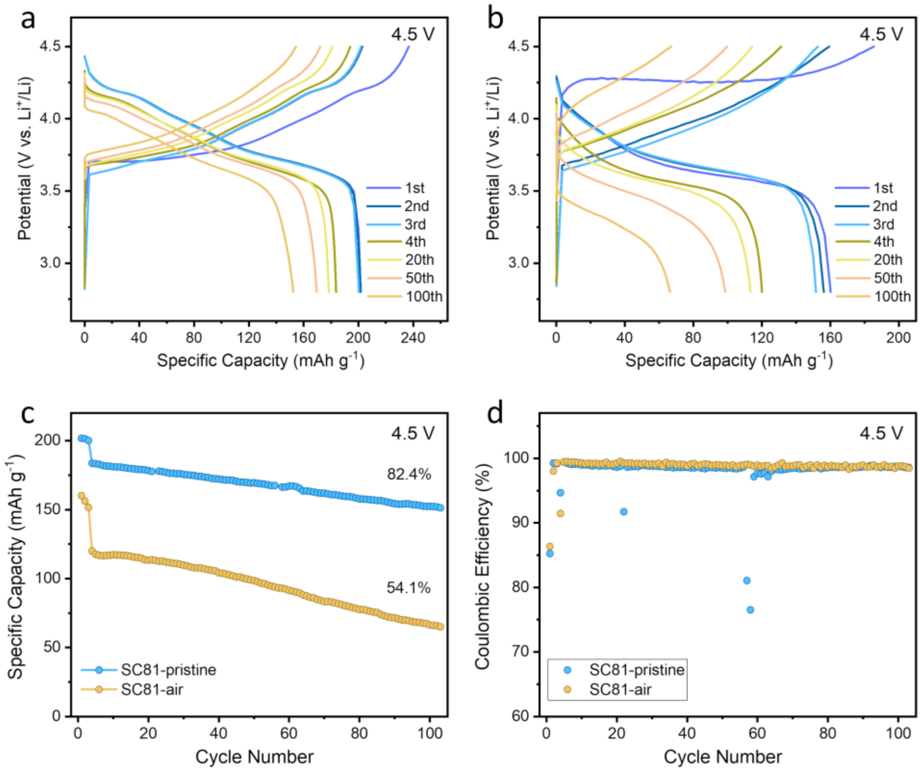

Supplymentary Fig. 9 | a, **b**, Another set of charge/discharge curves of pristine SC81 (a) and SC81-air (b) with voltage ranges of 2.8–4.5 V at 0.1C (20 mA g^{-1}) for first 3 formation cycles, and 0.5C (100 mA g^{-1}) for the rest cycles. **c**, **d**, Cyclic performance plot (c) and corresponding coulombic efficiency data (d) of pristine SC81 and SC81-air with voltage ranges of 2.8–4.5 V at 0.1C (20 mA g^{-1}) for first 3 formation cycles, and 0.5C (100 mA g^{-1}) for the rest cycles. These electrochemical tests were performed at a temperature of 25 ± 1 °C.

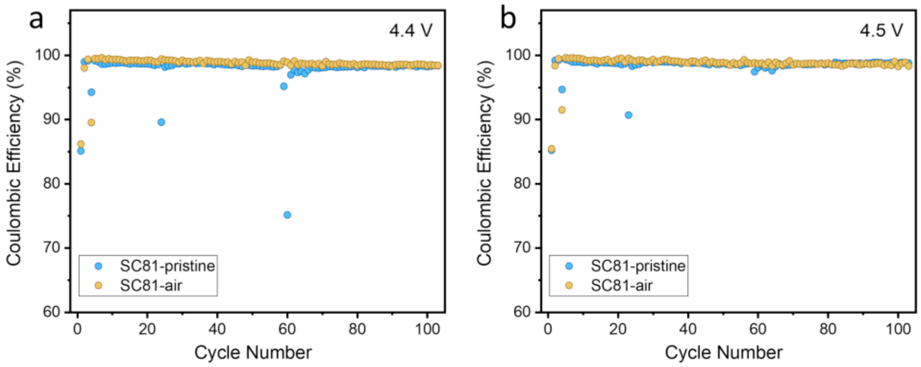

Supplymentary Fig. 10 | The corresponding coulombic efficiency data of Fig. 2e (a) and Fig. 2f (b). These electrochemical tests were performed at a temperature of 25 ± 1 °C.

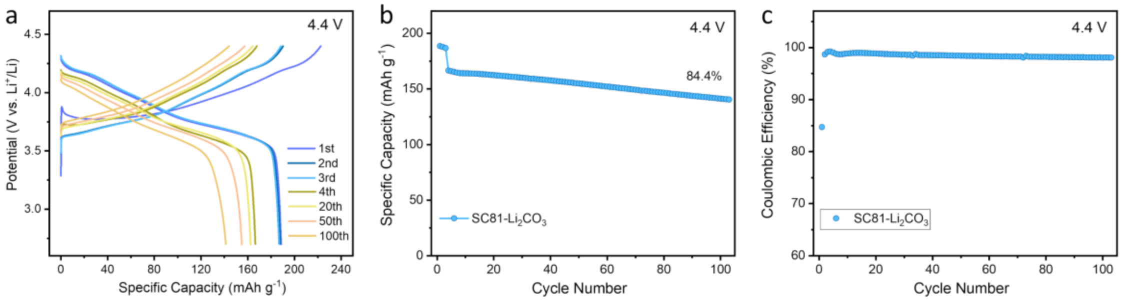

Supplymentary Fig. 11 | a-c, The charge/discharge curves (a), cycle performance plot (b), and corresponding coulombic efficiency data (c) of pristine SC81 mixed with 3.21% Li_2CO_3, with voltage ranges of 2.7–4.4 V at 0.1C (20mA g^{-1}) for first 3 formation cycles, and 0.5C (100mA g^{-1}) for the rest cycles. These electrochemical tests were performed at a temperature of 25 ± 1 °C.

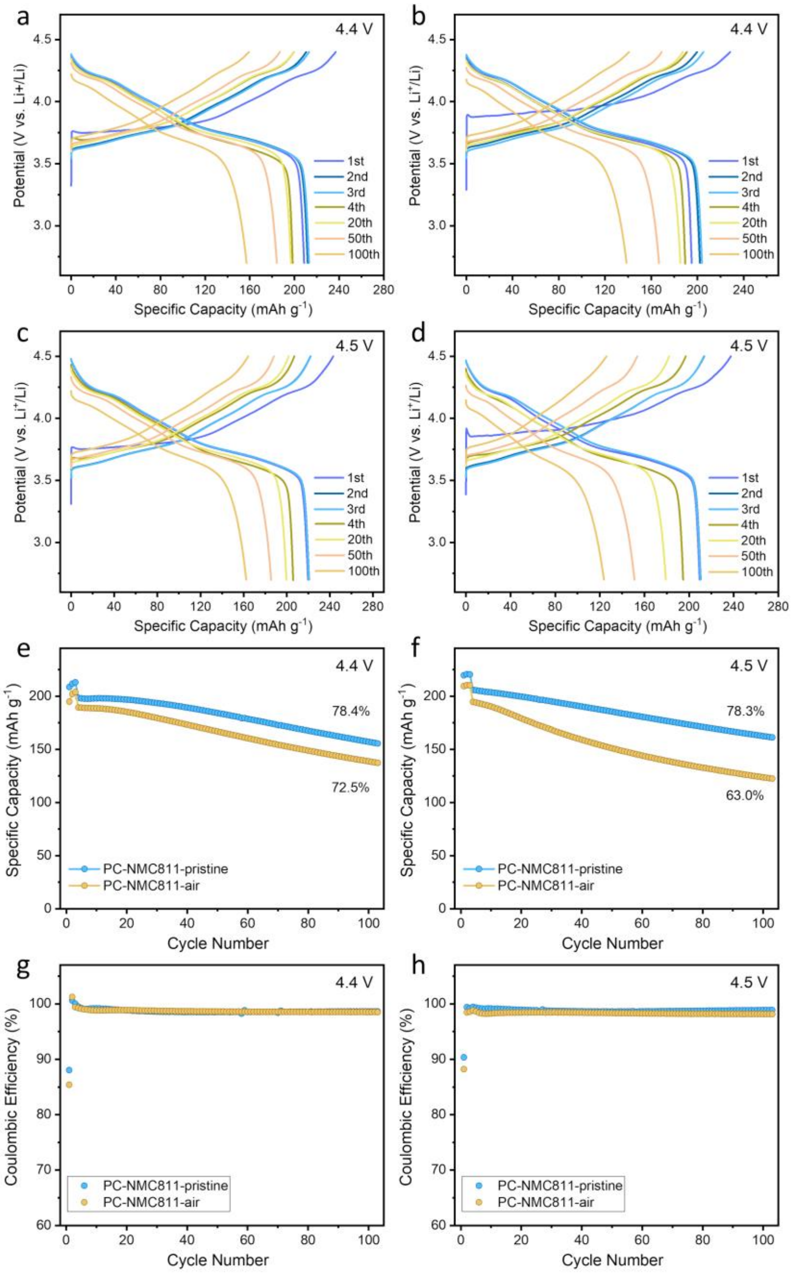

Supplymentary Fig. 12 | a, **b**, Charge/discharge curves of pristine PC-NMC811 (a) and PC-NMC811-air (b) with voltage ranges of 2.7–4.4 V at 0.1C (20 mA g^{-1}) for first 3 formation cycles, and 0.5C (100 mA g^{-1}) for the rest cycles. **c**, **d**, Charge/discharge curves of pristine PC-NMC811 (c) and PC-NMC811-air (d) with voltage ranges of 2.7–4.5 V at 0.1C (20 mA g^{-1}) for first 3 formation cycles, and 0.5C (100 mA g^{-1}) for the rest cycles. **e**, **f**, Cycle performance of pristine PC-NMC811 and PC-NMC811-air with a cutoff voltage of 4.4 V (e) and 4.5 V (f) at 0.1C (20 mA g^{-1}) for first 3 formation cycles, and 0.5C (100 mA g^{-1}) for the rest cycles. **g**, **h**, The corresponding coulombic efficiency data. These electrochemical tests were performed at a temperature of 25 ± 1 °C.

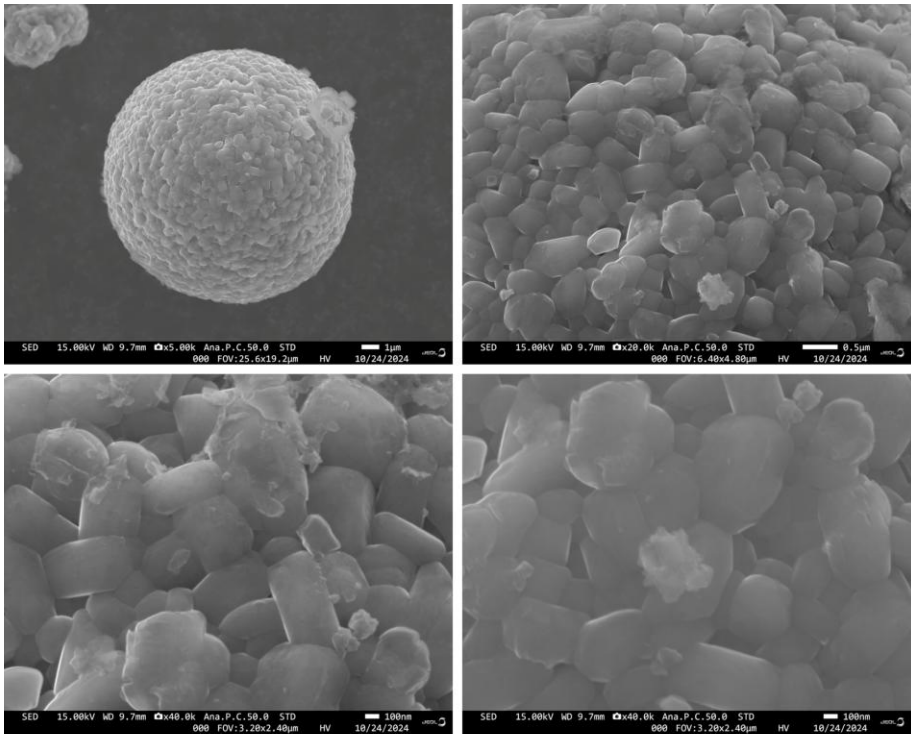

Supplymentary Fig. 13 | The SEM observations of the PC-NMC811-air particles, showing the residual lithium compounds on the surface of secondary particle.

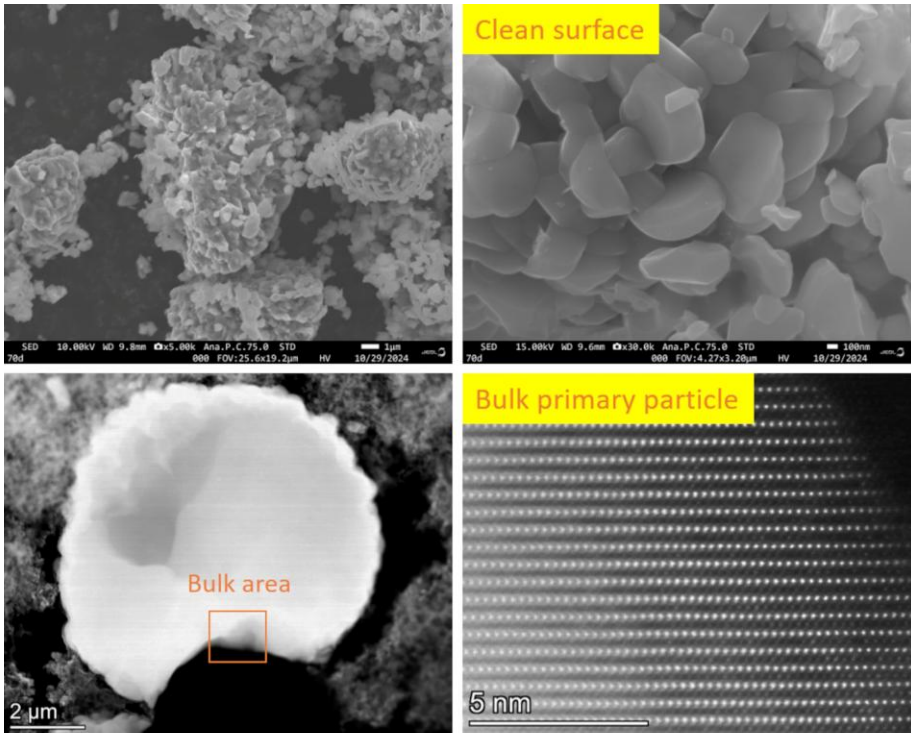

Supplymentary Fig. 14 | The SEM images of the crashed PC-NMC811-air particles. The inner primary particles show clean surfaces and no structural degradation.

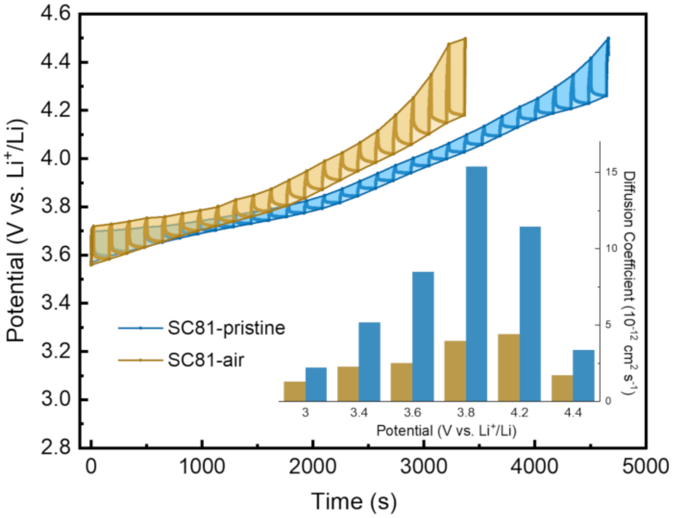

Supplymentary Fig. 15 | GITT curves of both samples during charging processes after formation cycles at 0.1C (20 mA g^{-1}). Inserted diagram: lithium ion diffusion coefficients of pristine SC81 and SC81-air samples as a function of voltage during Li extraction processes. These electrochemical tests were performed at a temperature of 25 ± 1 °C.

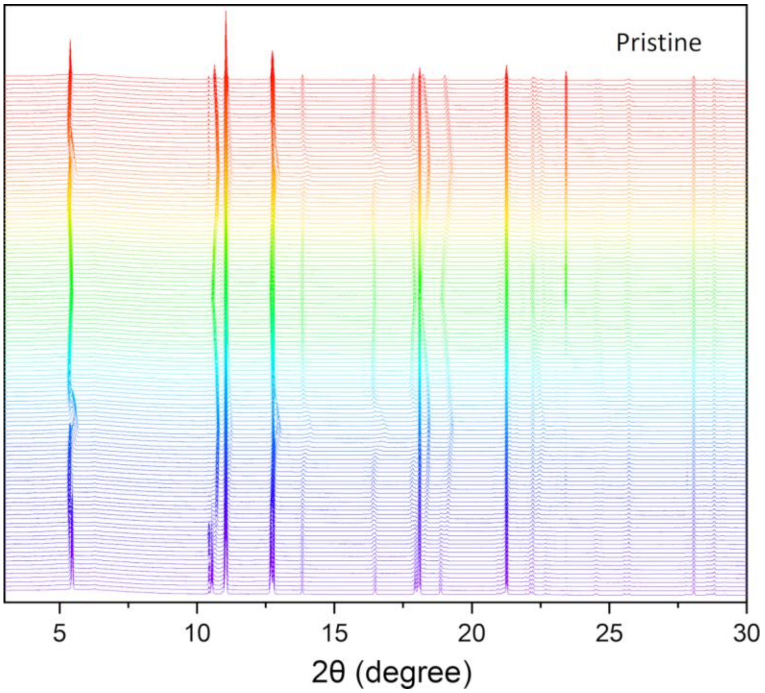

Supplymentary Fig. 16 | The in situ HEXRD patterns of the first two cycles for pristine SC81. The battery was performed at a specific current of 0.2C (40 mA g^{-1}) and a temperature of 25 ± 1 °C within the voltage ranges of 2.7–4.5 V. The obvious phase transitions and lattice parameter changes can be observed from in situ HEXRD pattern.

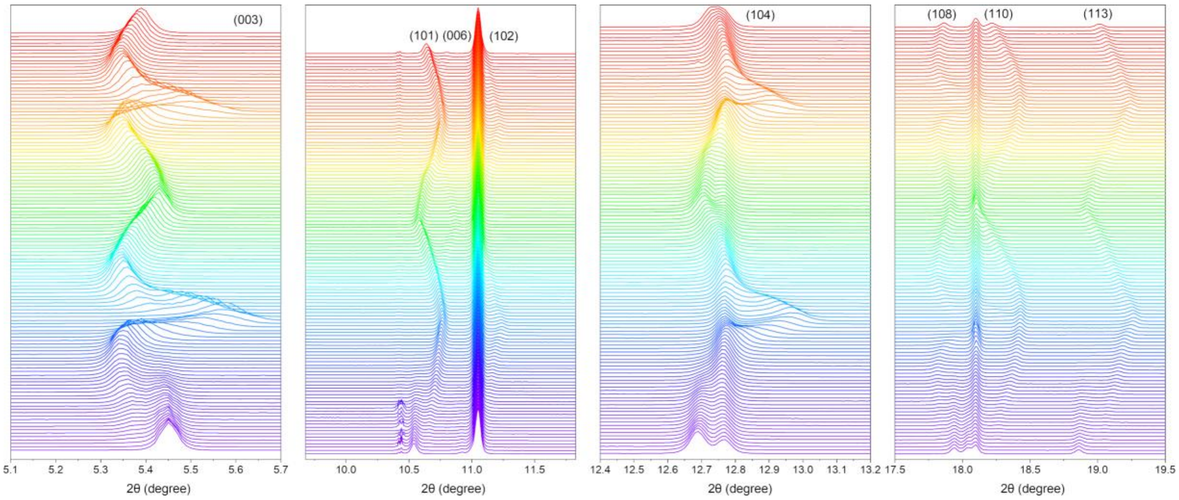

Supplymentary Fig. 17 | The enlarged XRD patterns of representative Bragg peaks of pristine SC81, including [003], [101], [006], [102], [104], [108], [110] and [113]. Some impurities in the XRD curve are identified as Al foil. The battery was performed at a specific current of 0.2C (40 mA g^{-1}) and a temperature of 25 ± 1 °C within the voltage ranges of 2.7–4.5 V.

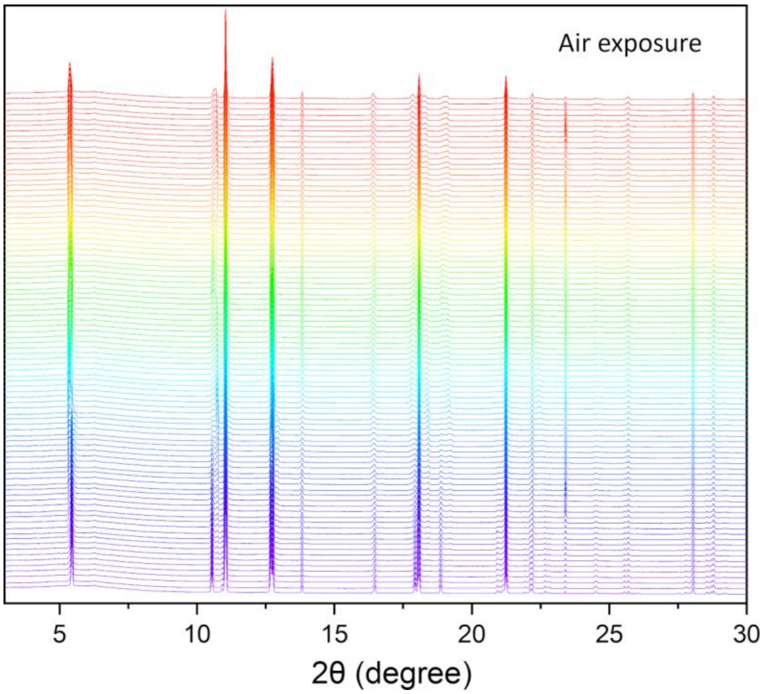

Supplymentary Fig. 18 | The in situ HEXRD patterns of the first two cycles for SC81-air. The battery was performed at a specific current of 0.2C (40 mA g^{-1}) and a temperature of 25 ± 1 °C within the voltage ranges of 2.7–4.5 V.

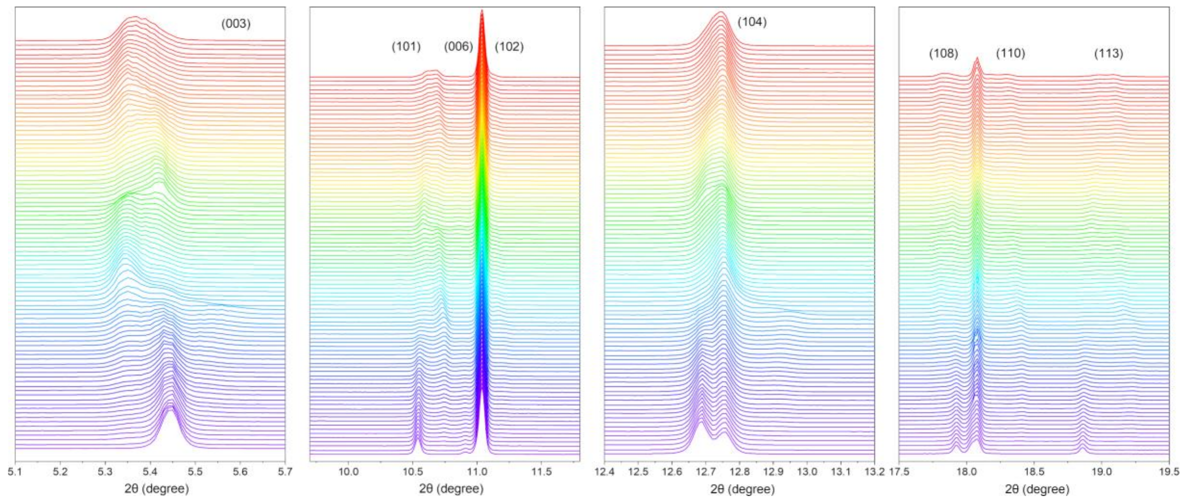

Supplymentary Fig. 19 | The enlarged XRD patterns of representative Bragg peaks of SC81-air, including [003], [101], [006], [102], [104], [108], [110] and [113]. Some impurities in the XRD curve are identified as Al foil. The battery was performed at a specific current of 0.2C (40 mA g^{-1}) and a temperature of 25 ± 1 °C within the voltage ranges of 2.7–4.5 V.

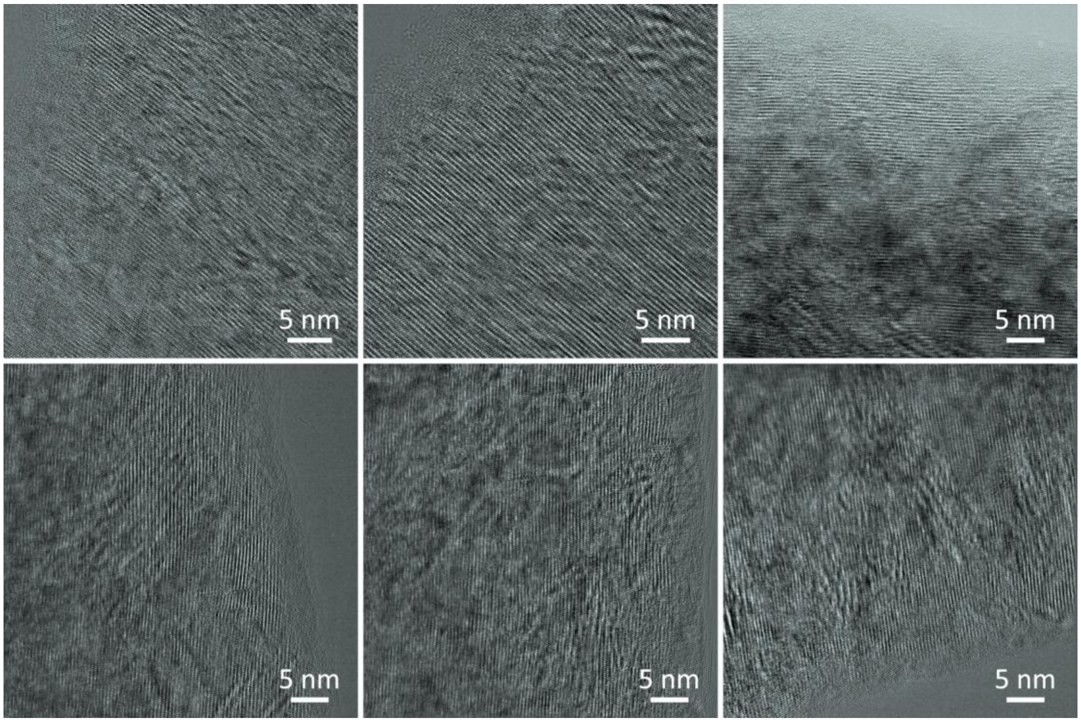

Supplymentary Fig. 20 | The HRTEM images of the SC81-air particle surfaces.

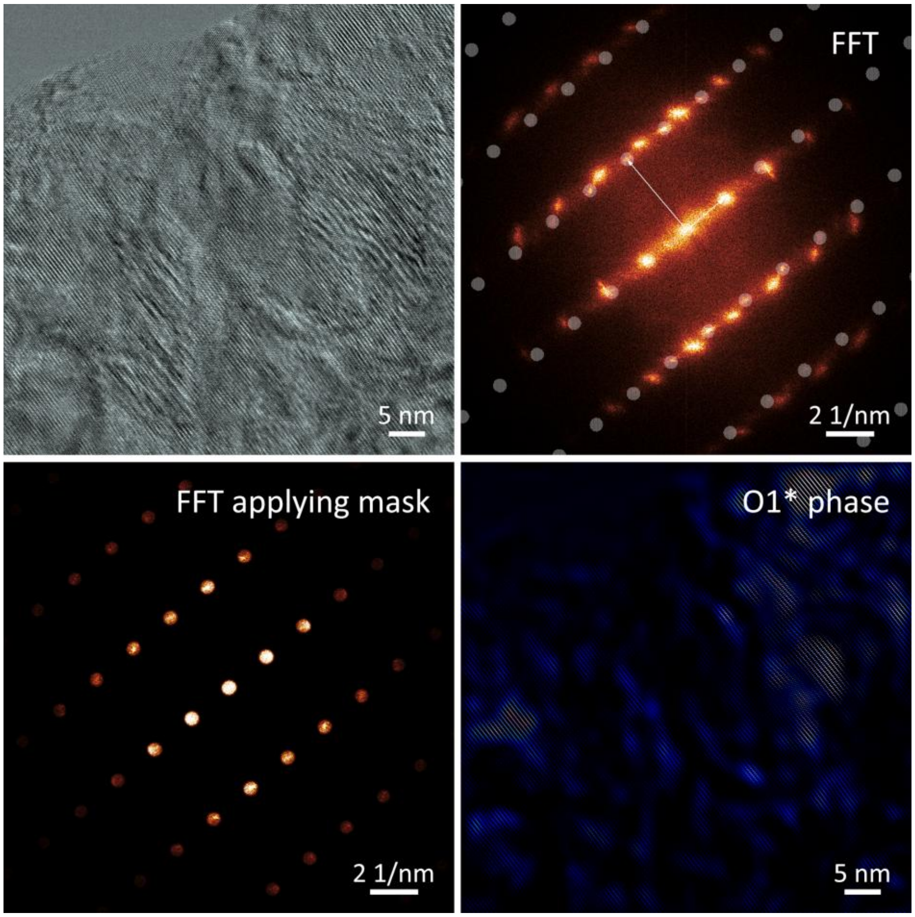

Supplementary Fig. 21 | Inverse FFT showing O1* pahse distribution by applying mask.

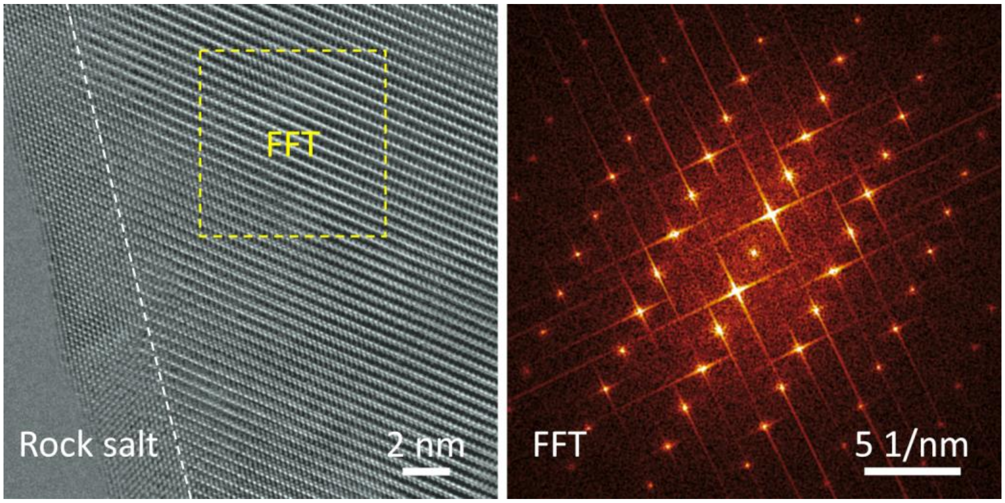

Supplymentary Fig. 22 | The HRTEM image and corresponding FFT pattern of the discharged SC81 positive electrode after 100 cycles at a specific current of 0.5C (100 mA g^{-1}) and a temperature of 25 ± 1 °C within the voltage ranges of 2.8–4.5 V.

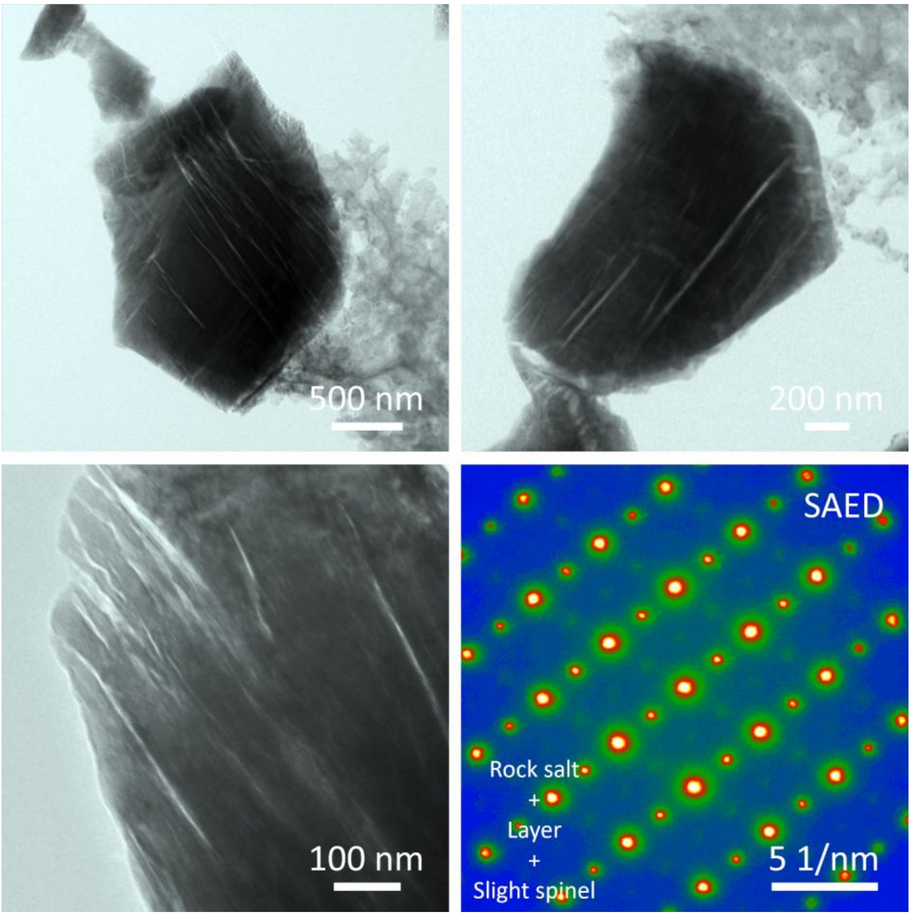

Supplymentary Fig. 23 |The TEM images of the discharged SC81-air positive electrode after 100 cycles at a specific current of 0.5C (100 mA g^{-1}) and a temperature of 25 ± 1 °C within the voltage ranges of 2.8–4.5 V, showing the intragranualr cracks in the single-crystyalline particles. The SAED pattern displaying the substantial transition of rock salt and spinel.

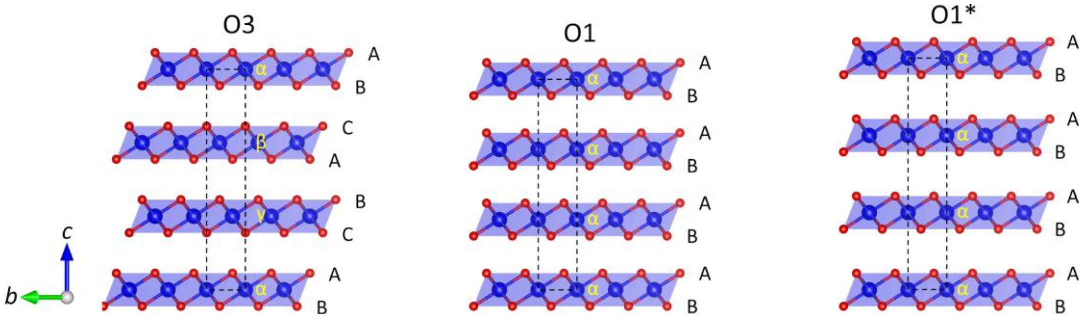

Supplementary Fig. 24 | Schematic diagram of the O3, O1, and O1* phases. The blue spheres denote Ni atoms, and the red spheres denote O atoms.

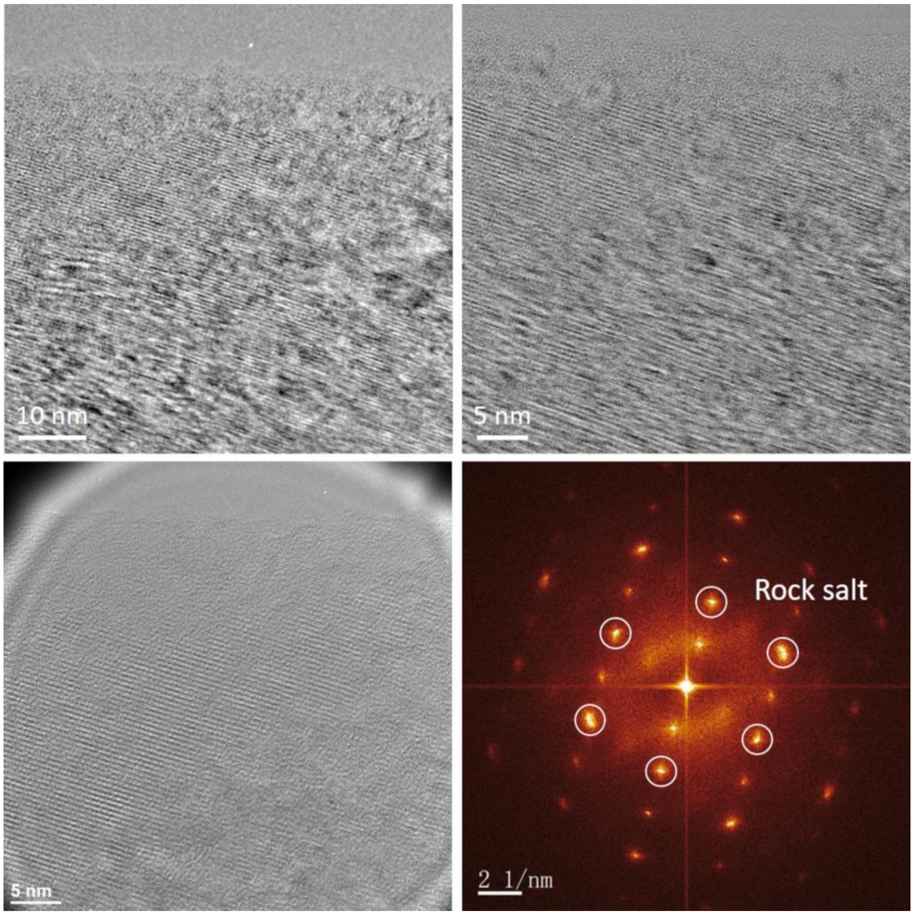

Supplementary Fig. 25 | Microscopic TEM characterization of the SC81-air surface under beam irradiation.
